# Dose-Escalated Salvage Radiotherapy for Macroscopic Local Recurrence of Prostate Cancer in the Prostate-Specific Membrane Antigen Positron Emission Tomography Era

**DOI:** 10.3390/cancers14194956

**Published:** 2022-10-10

**Authors:** Jörg Tamihardja, Leonie Zehner, Philipp E. Hartrampf, Sinan Cirsi, Sonja Wegener, Andreas K. Buck, Michael Flentje, Bülent Polat

**Affiliations:** 1 Department of Radiation Oncology, University of Wuerzburg, 97080 Wuerzburg, Germany; 2 Department of Nuclear Medicine, University of Wuerzburg, 97080 Wuerzburg, Germany

**Keywords:** prostate cancer, salvage radiotherapy, macroscopic recurrence, PSMA PET/CT, simultaneous integrated boost

## Abstract

**Simple Summary:**

Prostate cancer often relapses after initial radical prostatectomy, and salvage radiotherapy offers a second chance of cure for relapsed patients. Modern imaging techniques, especially prostate-specific membrane antigen positron emission tomography/computed tomography (PSMA PET/CT), enable radiation oncologists to target radiotherapy at the involved sites of disease. In a group of patients, PSMA PET/CT imaging can detect a macroscopic local recurrence with or without locoregional lymph node metastasis. In these cases, an escalation of the radiotherapy dose is often considered for controlling the visible tumor mass. As the evidence for dose-escalated salvage radiotherapy for macroscopic recurrent prostate cancer after PSMA PET/CT imaging is still limited, we address this topic in the current analysis. We found that the outcome of patients with dose-escalated salvage radiotherapy for macroscopic prostate cancer recurrence is encouragingly favorable, while the toxicity is very limited.

**Abstract:**

Background: The purpose of this study was to access the oncological outcome of prostate-specific membrane antigen positron emission tomography (PSMA PET/CT)-guided salvage radiotherapy (SRT) for localized macroscopic prostate cancer recurrence. Methods: Between February 2010 and June 2021, 367 patients received SRT after radical prostatectomy. Out of the 367 screened patients, 111 patients were staged by PSMA PET/CT before SRT. A total of 59 out of these 111 (53.2%) patients were treated for PSMA PET-positive macroscopic prostatic fossa recurrence. Dose-escalated SRT was applied with a simultaneous integrated boost at a median prescribed dose of 69.3 Gy (IQR 69.3–72.6 Gy). The oncological outcome was investigated using Kaplan-Meier and Cox regression analyses. The genitourinary (GU)/gastrointestinal (GI) toxicity evaluation utilized Common Toxicity Criteria for Adverse Events (version 5.0). Results: The median follow-up was 38.2 months. The three-year biochemical progression-free survival rate was 89.1% (95% CI: 81.1–97.8%) and the three-year metastasis-free survival rate reached 96.2% (95% CI: 91.2–100.0%). The cumulative three-year late grade 3 GU toxicity rate was 3.4%. No late grade 3 GI toxicity occurred. Conclusions: Dose-escalated PSMA PET/CT-guided salvage radiotherapy for macroscopic prostatic fossa recurrence resulted in favorable survival and toxicity rates.

## 1. Introduction

Salvage radiotherapy (SRT) for biochemical recurrence is a widely accepted therapy, offering a second chance to cure relapsed prostate cancer after radical prostatectomy [1,2]. While adjuvant radiotherapy may be considered in the presence of postoperative risk factors (mainly surgical margins and tumor size), early salvage radiotherapy is triggered by rising prostate-specific antigen (PSA) values. Salvage radiotherapy to the prostatic fossa is often applied without further delay in case of a biochemical recurrence for PSA values < 0.5 ng/mL. However, with advances in modern imaging techniques, the detection of macroscopic local recurrences is becoming more common, even for low PSA values [3]. In particular, prostate-specific membrane antigen positron emission tomography/computed tomography (PSMA PET/CT) offers high detection rates and has evolved rapidly to become the gold standard in the staging of biochemically relapsed patients [4,5,6]. Several studies indicate that pre-treatment PSMA PET/CT may lead to changes in target volume definition and disease management [7,8]. In the case of the PSMA PET/CT-based detection of macroscopic lesions on pre-SRT imaging, the target volumes may be adapted to ensure coverage of all visible locoregional disease sites. While the SAKK 09/10 trial [9] showed that a conventional dose of 64 Gy may offer sufficient biochemical control in biochemically relapsed patients, dose-escalation is often considered for visible macroscopic local recurrences [10]. Despite being an important clinical scenario, no consensus for dose-escalated radiotherapy for macroscopic recurrence in the prostate bed exists [11]. Moreover, as most retrospective studies for macroscopic prostate cancer recurrence have used conventional imaging or predominantly choline PET/CT imaging as a pre-SRT staging method, data from PSMA PET/CT-based SRT for macroscopic relapse are still limited at present [10,11,12,13,14,15,16,17]. In their analysis, Schmidt-Hegemann et al. did not observe a significant difference in biochemical relapse-free survival between 70 Gy for PET-positive local recurrences and 66 Gy for PET-negative recurrences, indicating the possibility of effective control of macroscopic disease by dose-escalation [18]. Moreover, Vogel et al. recorded encouraging data on the safety of PSMA PET/CT-based dose-escalated SRT versus conventional SRT for patients with recurrent prostate cancer [19]. Considering the lack of prospective data, we analyzed the oncological outcome and long-term toxicity of dose-escalated salvage radiotherapy for PSMA PET-positive macroscopic prostate cancer recurrence after radical prostatectomy. The current work adds to the body of evidence for PSMA PET/CT-based salvage radiotherapy and discusses the results of a dose-escalated single-center cohort in comparison with historical data.

## 2. Materials and Methods

Between February 2010 and June 2021, 367 patients were referred to our institution with biochemical relapse after radical prostatectomy. The institutional database was screened for PSMA PET-positive patients in pre-SRT PSMA PET/CT. In total, out of the 367 patients, 111 patients (30.2%) received PSMA PET/CT staging before initiation of salvage radiotherapy. A total of 21 (18.9%) patients were PSMA PET-negative, and 90 patients (81.1%) were PSMA PET-positive. Six (6.7%) patients suffered from PET-positive distant metastasis and received palliative radiotherapy. Twenty-five (27.8%) patients showed only PET-positive nodal involvement and received nodal salvage radiotherapy, which was the topic of earlier work [20]. The remaining 59 (65.6%) PSMA PET-positive patients showed a visible relapse in the prostatic fossa and received dose-escalated salvage prostatic fossa radiotherapy. In addition to prostatic fossa relapse, 11 of these 59 patients (18.6%) also had PSMA PET-positive locoregional lymph node involvement. This retrospective single-center analysis reports the oncological outcome and toxicity of these 59 patients who received SRT for locally relapsed prostate cancer.

The initial prostate cancer diagnosis was histologically proven in all patients, and the risk group classification according to D’Amico et al. was utilized [21]. Concomitant androgen deprivation therapy (ADT) was prescribed at the discretion of the treating urologist and recommended for patients with a Gleason score ≥ 8 or pre-SRT PSA values ≥ 0.7 ng/mL, in accordance with the German expert guidelines for ADT in biochemically recurrent prostate cancer [22]. The Gleason scores were derived from the prostatectomy pathology reports. In two cases, the postoperative Gleason score was classified as 3 + 2 before the International Society of Urological Pathology (ISUP) recommendations on the grading of prostate cancer were updated [23].

**Figure 1:**
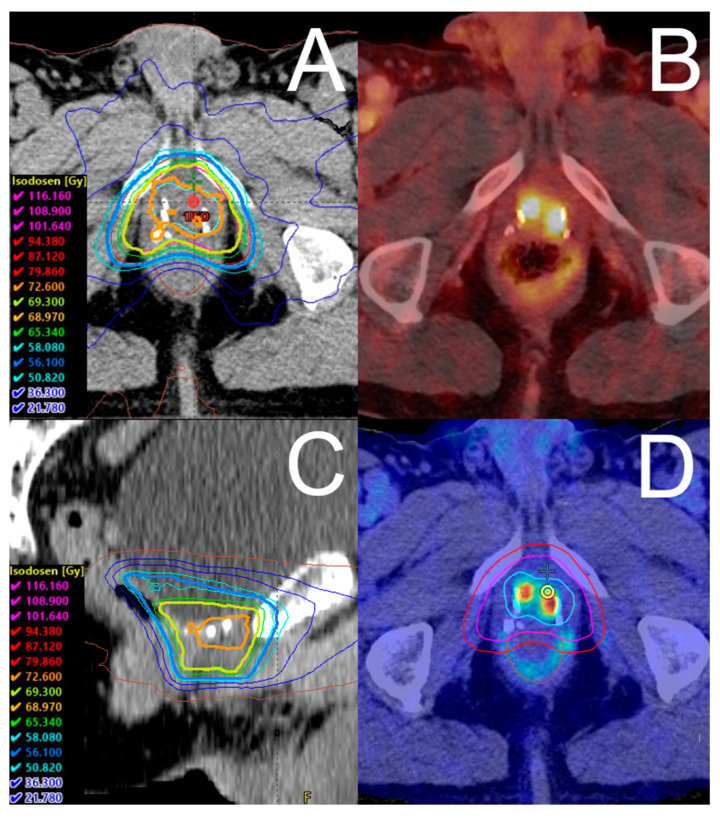
Exemplary dose distribution of salvage radiotherapy. Shown is an exemplary dose distribution in a patient receiving ^18^F-PSMA-1007 PET/CT-guided salvage radiotherapy in (**A**) axial view and (**C**) sagittal view. The corresponding ^18^F-PSMA-1007 PET/CT shows a PET-positive recurrence in the prostatic bed (**B**). The patient received volumetric modulated arc therapy with a simultaneous integrated boost to the local recurrence in three dose levels. (**D**) shows the fused PET/CT and corresponding radiotherapy contours. In 33 fractions, we prescribed single doses of 1.7 Gy for the PTV (red line), 2.1 Gy for the PTV_Boost1_ (pink line), and 2.2 Gy for the PTV_Boost2_ (turquoise line), resulting in total doses of 56.1 Gy (blue isodose), 69.3 Gy (bright green isodose), and 72.6 Gy (orange isodose), respectively.

Macroscopic local recurrence was defined as relapse visible in the PSMA PET/CT imaging. Before SRT, PSMA PET/CT imaging was conducted in 44 cases (74.6%) with ^68^Ga-PSMA I&T, and in 15 cases (25.4%) with ^18^F-PSMA-1007. In addition, magnetic resonance imaging (MRI) for radiotherapy planning was conducted in 37 patients (62.7%) before SRT, at the discretion of the treating radiation oncologist. The MRI protocol consisted of a native T2-weighted sequence, diffusion-weighted imaging with apparent diffusion coefficient mapping, and a contrast-enhanced T1-weighted sequence. The target volume delineation was based on the Radiation Therapy Oncology Group (RTOG) atlas for salvage prostate cancer and was adjusted at the discretion of the treating radiation oncologist for the institutional SIB concept. Pinnacle^3^ (Philips Radiation Oncology Systems, Fitchburg, WI, USA) was used as a treatment planning system (TPS). For target volume delineation, the PSMA PET/CT images and, if available, MR images were co-registered onto the primary planning CT image set within our TPS. Either intensity-modulated radiation therapy (IMRT) or volumetric modulated arc therapy (VMAT), both with simultaneous integrated boost (SIB), were used to deliver SRT. SRT was moderately hypofractionated, with 5 fractions per week, and cone-beam computed tomography-guided. Figure 1 illustrates an exemplary dose distribution.

The primary endpoint of this retrospective study was three-year biochemical progression-free survival. Biochemical progression was defined as a ≥0.5 ng/mL increase in PSA values over the PSA value nadir as defined by the GETUG AFU 16 trial [24]. Secondary endpoints were metastasis-free survival, overall survival, and gastrointestinal and genitourinary toxicity after three years of follow-up. Metastasis-free survival was defined as the time between the start of radiotherapy and the occurrence of imaging-based diagnosis of distant metastasis. The time between the start of radiotherapy and death from any cause was defined as overall survival. The follow-up was defined as the time between the start of radiotherapy and the date of the last follow-up. Physician-recorded toxicity was assessed at baseline, at the end of radiotherapy, six weeks after radiotherapy, and then every six months. Annual examinations were conducted after the first two years of follow-up. Common Terminology Criteria for Adverse Events (CTCAE) version 5.0 was used to assess the gastrointestinal (GI) and genitourinary (GU) side effects [25]. Toxicity events that occurred between the start of SRT and three months after SRT were recorded as acute toxicity. All later toxicity events were counted as late toxicity.

The Kaplan-Meier method, with the start of radiation therapy set as the baseline time, was used to generate three-year outcome estimates. Cox proportional hazards model analyses, considering as covariates Gleason score ≥ 8 (no/yes), concomitant ADT (no/yes), total dose to the local recurrence (continuous), time between radical prostatectomy and SRT (continuous), postoperative nodal status (pN0 versus pN1), postoperative surgical margin (R0 versus R1), and PSA value at the start of SRT (continuous), were performed to estimate the relative hazards of outcome parameters. Backward stepwise analysis selected Gleason score ≥ 8 (no/yes), the time between radical prostatectomy and SRT (continuous), and postoperative nodal status (pN0 versus pN1) were used as covariates for biochemical progression-free survival. For metastasis-free survival, Gleason score ≥ 8 (no/yes) and time between radical prostatectomy and SRT (continuous) were selected as the best-fitting covariates. R version 4.2.0 (The R Foundation, Vienna, Austria) was utilized for statistical analysis. All tests were two-sided, with statistical significance indicated by *p* < 0.05.

## 3. Results

The median follow-up was in total 38.2 months (IQR 29.0–48.3 months). Concomitant ADT was administered in 19 (32.2%) patients, with a median duration of 24.2 months (IQR 15.4–31.0 months). The patient characteristics are summarized in Table 1.

In total, seven cases (11.9%) relapsed biochemically, and six (10.2%) patients developed distant metastasis during follow-up. In six out of seven biochemically relapsed patients, a repeated PSMA PET/CT was conducted, detecting a local recurrence in one patient and no local recurrence in five patients after SRT. Distant metastasis manifested in two patients as bone metastasis and in four cases as non-regional lymph node metastasis. All six patients with distant metastasis after SRT received repeated PSMA PET/CTs, which detected no signs of local recurrence in the irradiated prostatic fossa. Overall, two patients (3.4%) died during follow-up.

**Figure 2:**
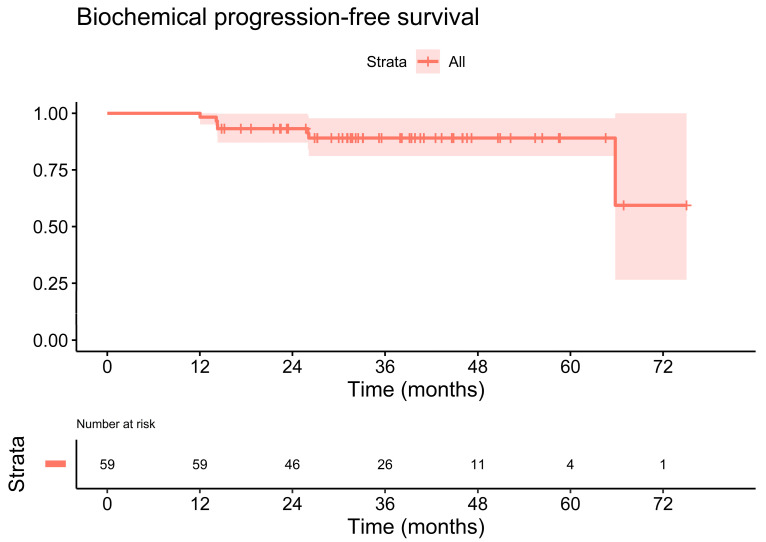
Kaplan-Meier plot of the biochemical progression-free survival. The estimated three-year biochemical progression-free survival rate was 89.1% (95% CI: 81.1–97.8%).

**Figure 3:**
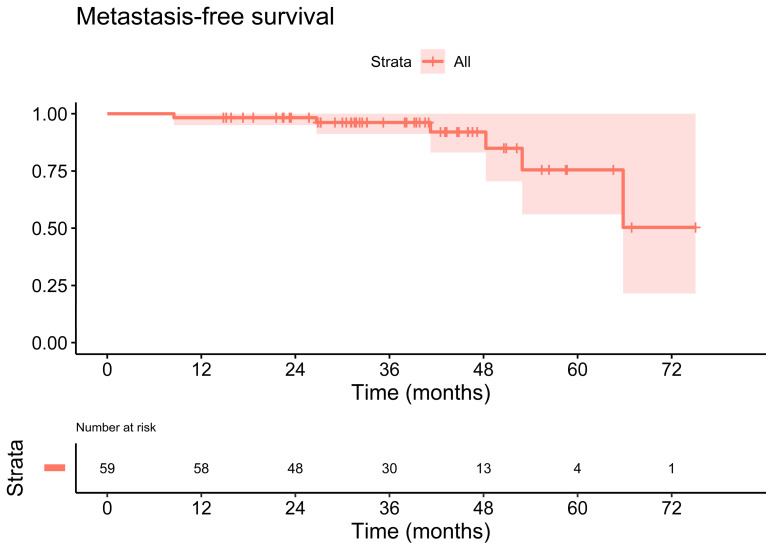
Kaplan-Meier plot of the metastasis-free survival. The estimated three-year metastasis-free survival rate was 96.2% (95% CI: 91.2–100.0%).

The estimated three-year biochemical progression-free survival rate was 89.1% (95% CI: 81.1–97.8%). Figure 2 illustrates the primary endpoint, biochemical progression-free survival, of the cohort. The estimated three-year metastasis-free survival rate reached 96.2% (95% CI: 91.2–100.0%), Figure 3. The three-year overall survival rate was 100.0% (95% CI: 100.0–100.0%).

Twenty-eight patients (47.5%) received an additional third dose level SIB (PTV_Boost2_) for macroscopic recurrence. The prescribed median PTV dose (D_95%_) was 56.1 Gy (inter-quartile range (IQR) 56.1–56.1 Gy) in 33 fractions (IQR 33–33 fractions) of 1.7 Gy per fraction (IQR 1.7–1.7 Gy). The prescribed median total PTV_Boost1_ dose (D_95%_) was 69.3 Gy (IQR 69.3–69.3 Gy), with a median dose per fraction of 2.1 Gy (IQR 2.1–2.1 Gy). The prescribed median total PTV_Boost2_ dose (D_95%_) was 72.6 Gy (IQR 72.6–75.5 Gy). In total, the median prescribed dose in the macroscopic lesion was 69.3 Gy (IQR 69.3–72.6 Gy) for all patients.

In 19 (32.2%) patients, the PSMA PET/CT showed a PET-positive lesion in the prostatic fossa, but a macroscopic lesion was not distinguishable on the CT scan. In 13 out of 19 PET-positive/CT-negative patients, an additional MRI was conducted. In 10 out of these 13 cases (76.9%), an MRI before SRT was able to show a visible lesion. In 3 out of 13 cases (23.1%), neither CT nor MRI could not detect a visible lesion. The median prescribed total dose was 69.3 Gy for PSMA PET-positive/CT-negative/MRI-negative cases. For PSMA PET-positive/CT-negative/MRI-positive cases, the median prescribed total dose was 71.0 Gy (IQR 69.3 Gy–72.6 Gy). Pre-SRT PSMA PET/CT imaging detected locoregional lymph node involvement in addition to the local recurrence in the prostatic fossa in 11 (18.6%) patients (see Table 1). In 96.6% of all patients, locoregional lymph node involvement was limited to zero to three PET-positive lymph nodes. Patients with PET-positive lymph nodes received irradiation of the macroscopic local recurrence, as described above, as well as dose-escalated radiotherapy of the pelvic lymph node metastases using the SIB technique. In total, the prescribed dose for the lymph node metastases (PTV_BoostLN_) was 66.9 Gy (IQR 61.4–69.3 Gy). The treatment characteristics are summarized in Table 2.

Uni- and multivariable Cox proportional hazards model analyses identified postoperative nodal status as a significant prognostic factor for biochemical progression-free survival (HR 9.11, 95% CI: 1.24–67.21, *p* = 0.030). For metastasis-free survival, Gleason score ≥ 8 (HR 19.25, 95% CI: 1.68–221.11, *p* = 0.018) as well as the time between radical prostatectomy and SRT (HR 1.03, 95% CI: 1.01–1.05, *p* = 0.019) were significant in multivariable Cox proportional hazards model analyses. The results of the uni- and multivariable Cox proportional hazards model analyses are summarized in Table 3.

Acute genitourinary side effects equal to or higher than CTCAE version 5.0 grade 2 were observed in 22.0% (n = 13), and acute gastrointestinal toxicity equal to or higher than grade 2 in 5.1% (n = 3). One patient developed grade 3 acute genitourinary toxicity (1.7%). Regarding late side effects, the cumulative three-year genitourinary toxicity equal to or higher than grade 2 amounted to 20.3% (n = 12), and the three-year late gastrointestinal toxicity equal to or higher than grade 2 to 1.7% (n = 1). The cumulative three-year late grade 3 genitourinary toxicity was 3.4% (n = 2) and consisted of two patients with urinary incontinence. Late grade 3 gastrointestinal toxicity did not occur.

## 4. Discussion

PSMA PET/CT-guided salvage radiotherapy for macroscopic local recurrence with or without pelvic lymph node metastasis after radical prostatectomy showed encouragingly high rates of biochemical progression-free survival, metastasis-free survival, as well as overall survival in this current analysis.

Up-to-date, prospective randomized trials assessing salvage radiotherapy for macroscopic prostate cancer recurrence are not yet available, while only a few retrospective studies address this topic (Table 4). Our outcome results compare favorably to published analyses: with a five-year biochemical progression-free survival rate of 89.1%, biochemical control is higher here than in the studies of Shelan et al., Bruni et al., Zili et al., and Zaine et al. [10,12,13,14]. A direct comparison of retrospective data is difficult due to the heterogeneity in patient characteristics and treatment parameters. Nonetheless, debatable factors influencing biochemical control are (a) the targeting of all sites of visible disease, (b) the patient characteristics, especially the pre-SRT PSA value level, (c) the usage of concomitant ADT, and (d) the applied dose-escalation concept.

First, biochemical control may have been influenced by PSMA PET/CT-staging in our study, which prohibited the inclusion of patients with multi-metastatic disease, who received systemic therapy instead. Evidence supporting the use of PSMA PET/CT as a pre-SRT staging method mainly stems from trials assessing PSMA PET/CT for patients with biochemical relapse. Schmidt-Hegemann reported the results of PSMA PET/CT-based SRT for biochemical recurrence and detected 30 cases of local recurrence in the prostatic fossa with or without pelvic lymph nodes [18]. After a median follow-up of 23 months, the biochemical recurrence-free survival was 78% in their analysis [18]. Meijer et al. showed improved oncological outcomes for patients who received pre-SRT PSMA PET/CT in biochemically recurrent prostate cancer: patients without PSMA PET/CT had a biochemical progression rate of 21% after one year, compared to 8% with pre-SRT PSMA PET/CT [26]. Moreover, Emmet et al. demonstrated the prognostic value of PSMA PET/CT for treatment response to SRT in patients with biochemical relapse. In particular, a negative PSMA PET/CT result predicted a high response rate to prostate fossa SRT [27]. In our analysis, 19 cases of CT negative but PSMA PET-positive lesions were observed, highlighting the superior diagnostic accuracy of PSMA PET/CT over conventional imaging [5]. Moreover, lymph node metastasis was detected in addition to the local recurrence in 18.6% of the patients by PSMA PET/CT, which subsequently led to an adjustment of the target volumes to cover all sites of visible disease with dose-escalated simultaneous integrated boost radiotherapy.

Second, the pre-SRT PSA value is known to be a significant predictor of biochemical control and metastatic disease progression [28]. Pre-SRT PSA value level did not significantly influence biochemical progression in our study, which might be due to the sample size. Nonetheless, the biochemical progression-free survival rate was favorable in our study, even though the average pre-SRT PSA value was rather high at 1.5 ± 2.3 ng/mL. Pre-SRT PSA value might be a surrogate marker for disease spread, and thorough staging with modern imaging naturally seems as though it would be important for discriminating local from advanced disease. Several studies on salvage radiotherapy for macroscopic prostate cancer recurrence did not employ, or only partly employed, PSMA PET/CT as pre-SRT staging [10,11,12,13,14,15,16,17]. Therefore, potential understaging in the mentioned trials may explain the difference in biochemical control compared with our study. Utilization of current imaging protocols is crucial in preventing understaging of disease and, therefore, target miss, which may have a negative impact on oncological outcome [29].

In the case of SRT for recurrent prostate cancer, the patients may benefit from additional hormonal therapy [24,30]. In the current analysis, ADT was administered in 32.2% of the patients concomitant with SRT, but it did not have a significant effect on biochemical progression-free survival or metastasis-free survival after a median follow-up of 38.2 months. Neither did ADT have a positive influence on outcome in the study conducted by Bruni et al., who reported a five-year biochemical progression-free survival of approximately 70% [12]. In comparison, Shelan et al. utilized short-term ADT in all patients, but biochemical progression-free survival was inferior with 44% after five years, which indicates that ADT may not be the only deciding factor for survival and disease control [10]. Locally recurrent prostate cancer could be a highly selected subgroup of recurrent prostate cancer, and patients may especially benefit from local SRT with or without additional ADT, as Bruni et al. suggested [12]. In our study, the time interval from radical prostatectomy to initiation of SRT was a median of 5.7 years. The long disease-free interval after primary surgery supports the hypothesis that the group of patients with locally recurrent prostate cancer might have had a favorable prognosis, while the treatment of fast progressing disease with unfavorable biology would benefit strongly from the addition of ADT. Further evidence in the form of data from randomized trials is needed to elucidate the role of ADT in salvage radiotherapy for macroscopic prostate cancer recurrence.

We escalated the radiation dose to the local recurrence up to a median total dose of 69.3 Gy, which is comparable to the studies of Zaine et al. and Lee et al. [13,15]. In primary prostate cancer, the FLAME trial demonstrated better biochemical control by dose-escalating the GTV up to 95 Gy [31]. In comparison, dose-escalation in our study was still relatively moderate, which may be the reason why the dose was not a significant prognostic factor in our analysis. This is in line with the published data on salvage radiotherapy for macroscopic local prostate cancer recurrence, as Bruni et al., Zilli et al., and Zaine et al. did not observe a benefit in biochemical control from higher doses [12,13,14]. For dose-escalation, most published studies utilized a sequential boost or a SIB at two dose levels [10,12,13,14,15]. The radiotherapy concept for macroscopic local recurrence at our institution included a SIB at up to three dose levels: PTV_Boost1_ encompassed the prostate bed and spared the rectum, while the highest dose, PTV_Boost2_, encompassed the macroscopic local recurrence without an additional margin. The low-dose PTV was generated by the application of a 10 mm margin around PTV_Boost1_ in all directions except posteriorly, where a 7 mm margin was used. The aim of this contouring concept is the sparing of the organs at risk, particularly the rectum, while enabling dose-escalation of the macroscopic recurrence. Higher doses per fraction may lead to increased toxicity, as Cozzarini et al. demonstrated for hypofractionated radiotherapy after radical prostatectomy [32]. Therefore, to avoid excessive toxicity, SRT was moderately hypofractionated in our series, with up to 2.2 Gy per fraction for the macroscopic recurrences. No cases of severe late gastrointestinal toxicity were observed. With two cases (3.4%) of late grade 3 urinary incontinence, genitourinary toxicity was acceptable. The toxicity rates in this analysis are comparable to other published studies. Zilli et al., for example, reported 7.3% grade 3–4 GU late toxicity and 1.8% late grade 3 GI toxicity with 64 Gy plus a 10 Gy boost, which represents the higher end of the spectrum [33]. Most other studies reported between zero and two cases of late grade 3 toxicity [10,12,13,15].

The inherent heterogeneity in patient characteristics attributable to the retrospective nature of the conducted analysis, as well as the relatively small number of patients, are limitations of our study. Another limitation is that patients who were not referred to our institution for salvage radiotherapy were not included in our database, and this may have biased the presented numbers for PSMA PET/CT-based detection of recurrences. Randomized data from prospective studies are needed to corroborate the high biochemical progression-free survival rate of dose-escalated salvage radiotherapy for macroscopic prostate cancer recurrence.

## 5. Conclusions

PSMA PET/CT-guided dose-escalated salvage radiotherapy with a simultaneous integrated boost to the local recurrence achieved encouragingly high rates of three-year biochemical progression-free survival, metastasis-free survival, and overall survival. Dose-escalated salvage radiotherapy in up to three dose levels led to effective disease control with low toxicity rates.

## Figures and Tables

**Table 1:** Patient characteristics.

Patient Characteristics	Overall
Patients, n (%)	59 (100.0)
Follow-up (months)	38.2 (29.0, 48.3)
KPS (%)	90 (90, 100)
Age at SRT Start (years)	71.1 (66.2, 76.3)
PSA at Primary Diagnosis (ng/mL)	6.9 (5.4, 11.6)
PSA at Primary Diagnosis, n (%)	
<10 ng/mL	33 (55.9)
10–20 ng/mL	12 (20.3)
>20 ng/mL	7 (11.9)
N/A	7 (11.9)
PSA at SRT Start (ng/mL)	0.8 (0.4, 1.7)
Gleason Score	
ISUP Group 1 (≤6)	15 (25.4)
ISUP Group 2 + 3 (7)	27 (45.8)
ISUP Group 4 + 5 (≥8)	16 (27.1)
N/A	1 (1.7)
Postoperative Tumor Classification n (%)	
pT2b	1 (1.7)
pT2c	29 (49.2)
pT3a	14 (23.7)
pT3b	13 (22.0)
N/A	2 (3.4)
Postoperative Nodal Status, n (%)	
Negative (pN0)	52 (88.1)
Positive (pN1)	5 (8.5)
N/A	2 (3.4)
Postoperative Surgical Margin, n (%)	
Negative (R0)	36 (61.0)
Positive (R1)	16 (27.1)
N/A	7 (11.9)
Initial D’Amico Risk Class, n (%)	
High	57 (96.6)
N/A	2 (3.4)
Imaging Method, n (%)	
PSMA PET/CT	59 (100.0)
MRI	37 (62.7)
Disease Pattern Before SRT, n (%)	
Prostatic fossa recurrence	48 (81.4)
Prostatic fossa recurrence + locoregional LN metastasis	11 (18.6)
Number of LN Metastases, n (%)	
0	48 (81.4)
1	4 (6.8)
2	3 (5.1)
3	2 (3.4)
≥4	2 (3.4)
Time from Surgery to PSMA PET/CT (months)	67.0 (37.4, 117.9)
Time from Surgery to SRT (months)	68.1 (38.8, 119.3)

Abbreviations: ISUP = International Society of Urological Pathology; KPS = Karnofsky performance score; LN = lymph node; MRI = magnetic resonance imaging; N = number; N/A = not available; PET = positron emission tomography; PSA = prostate-specific antigen; PSMA = prostate-specific membrane antigen; SRT = salvage radiotherapy. Numbers are noted as median (quartile 1, quartile 3) or frequency (percentage).

**Table 2:** Treatment characteristics.

Treatment Characteristics	Overall
Concomitant ADT, n (%)	
ADT	19 (32.2)
No ADT	40 (67.8)
ADT duration (months)	24.2 (15.4, 31.0)
SIB concept, n (%)	
Two dose levels	31 (52.5)
Three dose levels	28 (47.5)
Number of fractions	33 (33, 33)
Prostate bed PTV dose (Gy)	56.1 (56.1, 56.1)
Prostate bed PTV_Boost1_ dose (Gy)	69.3 (69.3, 69.3)
Prostate bed PTV_Boost2_ dose (Gy)	72.6 (72.6, 75.5)
Lymph node PTV_BoostLN_ (Gy)	66.9 (61.4, 69.3)

Abbreviations: ADT = androgen deprivation therapy; LN = lymph node; N = number; PTV = planning target volume; RT = radiotherapy; SIB = simultaneous integrated boost; SRT = salvage radiotherapy. Numbers are noted as median (quartile 1, quartile 3) or frequency (percentage).

**Table 3:** Uni- and multivariable Cox proportional hazards model analyses.

**Biochemical Progression-Free Survival**	**Univariable**		**Multivariable**	
**Variables**	**HR (95% CI)**	*p* Value *	**HR (95% CI)**	*p* Value *
PSA at SRT Start (ng/mL)	1.05 (0.80, 1.38)	0.743		
Total Dose (Gy)	1.08 (0.79, 1.48)	0.644		
Time from RP to SRT (months)	1.00 (0.99, 1.02)	0.551	1.02 (1.00, 1.04)	0.101
Gleason Score ≥ 8				
No	Ref		Ref	
Yes	2.95 (0.59, 14.65)	0.186	11.44 (0.65, 202.39)	0.096
Concomitant ADT				
No	Ref		Ref	
Yes	2.33 (0.47, 11.57)	0.300		
Postoperative Nodal Status				
Negative (pN0)	Ref		Ref	
Positive (pN1)	8.65 (1.50, 50.09)	0.016	9.11 (1.24, 67.21)	0.030
Postoperative Surgical Margin				
Negative (R0)	Ref		Ref	
Positive (R1)	0.55 (0.06, 4.95)	0.596		
**Metastasis-free survival**	**Univariable**		**Multivariable**	
**Variables**	**HR (95% CI)**	*p* Value *	**HR (95% CI)**	*p* Value *
PSA at SRT Start (ng/mL)	1.06 (0.73, 1.56)	0.752		
Total Dose (Gy)	1.13 (0.76, 1.67)	0.533		
Time from RP to SRT (months)	1.00 (0.99, 1.02)	0.541	1.03 (1.01, 1.05)	0.019
Gleason Score ≥ 8				
No	Ref		Ref	
Yes	5.31 (0.85, 33.08)	0.074	19.25 (1.68, 221.11)	0.018
Concomitant ADT				
No	Ref		Ref	
Yes	1.37 (0.23, 8.22)	0.730		
Postoperative Nodal Status				
Negative (pN0)	Ref		Ref	
Positive (pN1)	3.09 (0.34, 28.53)	0.319		
Postoperative Surgical Margin				
Negative (R0)	Ref		Ref	
Positive (R1)	0.03 (0.00, 487.93)	0.481		

* Likelihood ratio test. Bold *p* values indicate significant results. Abbreviations: ADT = androgen deprivation therapy; CI = confidence interval; HR = hazard ratio; PSA = prostate-specific antigen; Ref = reference; RP = radical prostatectomy; SRT = salvage radiotherapy.

**Table 4:** Comparative literature overview.

	Number of pts	RT Dose (Gy)	Median Follow-Up	bPFS	mPFS	OS
Shelan [10]	69	72–74 Gy	38 mo	5 y: 44%	5 y: 76%	NA
Bruni [12]	105	>70 Gy: 58 pts 66–70 Gy: 43 pts < 66 Gy: 4 pts	52 mo	5 y: 69.7%	5 y: 86.1%	5 y: 85.5%
Zili [14]	131 *	64–74 Gy	36 mo	5 y: 45.6%	5 y: 85.2%	5 y: 92.5%
Zaine [13]	89	Median 70 Gy	54 mo	5 y: 50.8%	5 y: 76.6%	5 y: 90.2%
Lee [15]	60	Median 70.2 Gy	83 mo	7 y: 67.0%	7 y: 83.6%	7 y: 91.2%
Schmidt-Hegemann [18]	30 **	Median 70 Gy	23 mo	2 y: 78.0%	-	-
Our series	59	Median 69.3 Gy	38 mo	3 y: 89.1%	3 y: 96.2%	3 y: 100.0%

Abbreviations: mo = months; y = years; bPFS = biochemical progression-free survival; mPFS = metastasis-free survival; OS = overall survival; pts = patients; RT = radiotherapy. * 131 out of 171 patients with local recurrence. ** 30 out of 90 patients with local recurrence.

## Data Availability

The data presented in this study are available on request from the corresponding author. The data are not publicly available due to privacy restrictions.
